# Complete genome sequences of *Ruminococcus torques* strains JCM 36208 and JCM 36209, isolated from the feces of a healthy Japanese male

**DOI:** 10.1128/MRA.00632-23

**Published:** 2023-10-06

**Authors:** Atsushi Hisatomi, Dieter M. Tourlousse, Mayu Hamajima, Moriya Ohkuma, Yuji Sekiguchi, Mitsuo Sakamoto

**Affiliations:** 1 Microbe Division/Japan Collection of Microorganisms, RIKEN BioResource Research Center, Tsukuba, Ibaraki, Japan; 2 Biomedical Research Institute, National Institute of Advanced Industrial Science and Technology (AIST), Tsukuba, Ibaraki, Japan; Wellesley College, Wellesley, Massachusetts, USA

**Keywords:** human microbiome, isolate, genome sequence, *Ruminococcus torques*

## Abstract

Here, we report the complete genome sequences of two *Ruminococcus torques* strains (JCM 36208 and JCM 36209) that were newly isolated from the feces of a healthy Japanese male. Both genomes consist of a single circular chromosome with a length of ~2.8 Mbp and a G+C content of 41.8%.

## ANNOUNCEMENT

*Ruminococcus torques* is a Gram stain-positive, obligately anaerobic bacterium. The members of the genus *Ruminococcus* was found in the human gut (1, 2). *R. torques* is known as a bile acid-converting bacterium (3). Here, we present complete genomes of two *R. torques* strains isolated from the feces of a 30-year-old healthy Japanese male.

For isolation, serial dilutions of a freshly collected fecal sample in pre-reduced phosphate-buffered saline (PBS) were plated onto Columbia blood agar or Eggerth-Gagnon (EG) agar (Nissui) supplemented with 5% (vol/vol) horse blood. Two strains, designated 17CBH32 (=JCM 36208) and 17EGH124 (=JCM 36209), were isolated after incubation at 37℃ for 1 to 2 days under an atmosphere of H_2_/CO_2_/N_2_ (5/5/90, vol/vol/vol). The grown colonies, purified by streaking, were identified by near-full-length 16S rRNA gene sequencing, using universal primers 27F and 1492R for amplification (4). JCM 36208 and JCM 36209 share 16S rRNA gene similarity with the type strain of *R. torques* (accession number AB910746; 99.8% and 99.6% identity, respectively). Each strain was grown on five EG plates to prepare the genomic DNA. Genomic DNA was extracted using the MagAttract HMW DNA Kit (Qiagen) following the manufacturer’s provided instructions. Libraries for sequencing were constructed with ONT’s Native Barcoding Kit 24 (SQK-NBD114.24), without fragmentation and/or size selection, and sequenced on an R10.4.1 flow cell with a GridION device. All bioinformatics analyses were run with default parameters unless indicated. Bases were called with Dorado (v.0.2.1, https://github.com/nanoporetech/dorado), specifying model dna_r10.4.1_e8.2_400bps_sup@v.4.0.0 and option -emit-fastq. Duplex Tools (v.0.3.1, https://github.com/nanoporetech/duplex-tools), run for four iterations with option -allow_multiple_splits, was used to identify and split the chimeric and/or concatenated reads. Guppy’s guppy_barcoder command (v.6.4.6, with options -enable_trim_barcodes -barcode_kits SQK-NBD114-24) was used for demultiplexing. After removal of short (<1,000 bases) and low-quality (average quality score of <12) reads using NanoFilt (v.2.8.0) (5), high-quality reads with an estimated coverage of ~150× were retrieved using Filtlong (v.0.2.0, https://github.com/rrwick/Filtlong), with option -mean_q_weight 10. This retained 71,529 reads (419,134,763 bp with an N50 of 9,003 bp) and 100,297 reads (419,411,335 bp with an N50 of 5,564 bp) for JCM 36208 and JCM 36209, respectively. Genome assemblies were generated using Flye (v.2.9.1) (6), yielding a single contig, marked as circular by Flye, for each strain. The contigs were finally polished using Medaka (v1.7.3, https://github.com/nanoporetech/medaka), specifying model r1041_e82_400bps_sup_v.4.0.0. Completeness and contamination of the genome were assessed using CheckM (v.1.1.3), lineage_wf (7), and annotated with the DDBJ Fast Annotation and Submission Tool (v.0.5.5) (8).

Both genome assemblies have a completeness of 98.5% and contamination of 0.0% and consist of a single circular chromosome with a length of ~2.8 Mbp and a G+C content of 41.8% (Table 1). More information is shown in Table 1.

**TABLE 1:** Summary of DNA sequence assembly data

	Data for strain	
Feature	JCM 36208	JCM 36209
Genome size (bp)	2,794,081	2,796,037
GC content (%)	41.8	41.8
No. of tRNAs	50	50
No. of rRNAs	12	12
Coverage (x)^*a*^	152	150
No. of CDSs^*b*^	2,638	2,589
Accession no.	AP028248	AP028249

^*a*^ Coverage after subsampling of length- and quality-filtered reads using Filtlong (see text for details).

^*b*^ CDSs, coding sequences.

## Data Availability

The complete genome sequences were deposited in DDBJ/ENA/GenBank under accession numbers AP028248 (=JCM 36208) and AP028249 (=JCM 36209). Sequence reads are available in the DDBJ Sequence Read Archive under accession numbers DRR493111 and DRR493112.
